# Health technology assessment and reimbursement policy for oncology orphan drugs in Central and Eastern Europe

**DOI:** 10.1186/s13023-020-01556-9

**Published:** 2020-10-08

**Authors:** Krzysztof Piotr Malinowski, Paweł Kawalec, Wojciech Trąbka, Christoph Sowada, Guenka Petrova, Manoela Manova, Alexandra Savova, Pero Draganić, Juraj Slabý, Agnes Männik, Kristóf Márky, Zinta Rugaja, Jolanta Gulbinovic, Tomas Tesar, Marian Sorin Paveliu

**Affiliations:** 1 Institute of Public Health, Faculty of Health Sciences, Jagiellonian University Medical College, ul. Grzegórzecka 20, 31-531 Kraków, Poland; 2 Bioinformatics and Public Health Department, Faculty of Medicine and Health Sciences, Andrzej Frycz Modrzewski Krakow University, Kraków, Poland; 3 Faculty of Pharmacy, Medical University of Sofia, Sofia, Bulgaria; 4 National Council On Prices and Reimbursement of Medicinal Products, Sofia, Bulgaria; 5 Agency for Medicinal Products and Medical Devices of Croatia, Zagreb, Croatia; 6 Department of Biotechnology, University of Rijeka, Rijeka, Croatia; 7 Section of Pricing and Reimbursement Regulation, State Institute for Drug Control, Prague, Czechia; 8 Institute of Family Medicine and Public Health, University of Tartu, Tartu, Estonia; 9 National Institute of Health Insurance Fund Management, Budapest, Hungary; 10 The National Health Service, Riga, Latvia; 11 Department of Pathology, Forensic Medicine and Pharmacology, Faculty of Medicine, Institute of Biomedical Sciences, Vilnius University, Vilnius, Lithuania; 12 Department of Organisation and Management in Pharmacy, Faculty of Pharmacy, Comenius University in Bratislava, Bratislava, Slovakia; 13 Titu Maiorescu University, Bucharest, Romania

**Keywords:** Orphan drugs, Oncology, Reimbursement, Health technology assessment, Policy, Central and Eastern Europe

## Abstract

**Background:**

The reimbursement of orphan drugs (OD) is an increasingly important for country policymakers, and still insufficiently understood, especially in Central and Eastern Europe. The aim of this research was to provide a comprehensive description of country-specific health technology assessment (HTA) policies as well as evaluate the percentage of HTA recommendations and reimbursement decisions for oncology OD. In addition, the study was designed to elucidate the impact of reimbursement of these drugs on the public budget and the agreement between HTA recommendations and reimbursement decisions in the analysed countries. A questionnaire survey was used to collect data on the reimbursement status, HTA recommendation, marketing authorisation, and public expenses on reimbursement in 2014, 2015, and 2016 for all oncology drugs with an orphan designation by the European Medicine Agency in 2017 in Bulgaria, Croatia, Czechia, Estonia, Hungary, Latvia, Lithuania, Poland, Romania, and Slovakia. The agreement between the HTA recommendation and reimbursement status was assessed using the kappa coefficient. The Pearson’s correlation was used to analyse the relationship between gross domestic product (GDP) and GDP per capita and reimbursement expenses.

**Results:**

A total of 36 drugs were analysed (25% conditionally approved; 5.56% approved under exceptional circumstances). The share of reimbursed drugs ranged from 11.11% in Latvia to 41.67% in Poland. The highest share of positive recommendations was observed for Bulgaria and Estonia (36.11%), and the lowest, for Latvia (11.11%). The agreement varied from 0.4 for Poland to 1 for Latvia, Hungary, and Slovakia. Expenses were correlated with GDP (0.95 [0.81–0.99]), and not with GDP per capita (0.54 [− 0.136 to 0.873]). Expenses per capita were not correlated with GDP per capita (0.52 [− 0.15 to 0.87]).

**Conclusions:**

In Hungary, Latvia, and Slovakia, a positive recommendation was associated with a reimbursement, and a negative one, with the lack of reimbursement. The reimbursement of oncology OD is associated with a growing burden for public budget, and the expenses are correlated with the total GDP. The highest share of drugs with any recommendation was observed in Poland, and the lowest, in Latvia and Romania. The share of reimbursed drugs was the lowest in Latvia and the highest in Poland.

## Background

Rare diseases mostly include inherited life-threatening or chronically debilitating diseases that affect fewer than 5 of 10,000 people, according to the definition developed by the European Medicines Agency (EMA). Oncological diseases constitute around one-third of rare diseases and include, for example, lymphoblastic leukaemia, CD30 + Hodgkin lymphoma, or advanced soft tissue sarcoma [1–5]. The reimbursement of those drugs—called oncology orphan drugs—is the main way of making them accessible for patients with rare oncological diseases.

The EMA approves orphan drugs through a centralised procedure and issues the orphan designation; however, the status for particular drugs varies between countries. Orphan drugs can be approved conditionally (if the required clinical data regarding, for example, safety and efficacy are not yet available but will become available within a specified period of time) or under exceptional circumstances (if the required data regarding, for example, safety will never be accessible, for example, due to ethical concerns) [6, 7].

Recommendations and the final reimbursement decisions made by health technology assessment (HTA) agencies in specific countries are particularly interesting for decision-makers in Central and Eastern Europe (CEE) because orphan drugs, in general, seem to be one of the most expensive pharmaceuticals [8]. It is becoming increasingly popular in CEE countries to implement special regulations regarding the reimbursement of orphan drugs, which affects not only the decision-making process but also the procedures of developing HTA recommendations and requirements [5]. The reimbursement of orphan drugs is the main determinant of patients’ access to innovative therapies, with the better availability of treatments in western European countries, as shown by German and French research [5, 14]. However, no sub-analysis for oncology orphan drugs was performed in relation to the CEE countries which are characterised by limited access to orphan biotechnological drugs, with Macedonia and Estonia having only one drug reimbursed, followed by Romania and Serbia with two drugs reimbursed, Bulgaria with three drugs reimbursed, Slovakia with four drugs reimbursed, and Croatia with seven drugs reimbursed [16].

To fill the knowledge gap, we aimed to comprehensively review the process of developing the recommendations based on HTA practices in these countries as well as to assess and compare the percentage of HTA recommendations and reimbursement status for oncology orphan drugs in selected CEE countries. In addition, we aimed to assess an agreement between recommendations and reimbursement status within the CEE countries for all orphan drugs that were assessed in selected countries, as this would provide information on relations between HTA assessment and the final reimbursement decision. Finally, we aimed to evaluate the expenditures from the public budget on the reimbursement of oncology orphan drugs over the period of 3 years, from 2014 to 2016.

## Results

We analysed 36 oncology drugs with orphan designation in 2017. Nine of them (25%) received conditional approval by the EMA and only 2 (5.56%) were approved under exceptional circumstances (Table 1).

**Table 1:** Reimbursement status and recommendations issued for analysed drugs in 2017

Medicine name	Active substance	Approval type	Recommendation/reimbursement In analysed countries									
			Bulgaria	Croatia	Czechia	Estonia	Hungary	Latvia	Lithuania	Poland	Romania	Slovakia
Adcetris	Brentuximab vedotin	Conditional approval	✓/✓	✓/✓	./✗	✓/✓	✓/✓	./✗	./✗	✓/✓	./✗	./✗
Arzerra	Ofatumumab	Unconditional	✓/✓	✓/✓	./✗	./✗	✗/✗	./✗	./✗	✗/✗	✓/✓	✓/✓
Atriance	Nelarabine	Exceptional circumstances	✓/✓	./✗	✓/✗	./✗	✓/✓	✗/✗	./✗	✓/✓	✓/✓	./✗
Blincyto	Blinatumomab	Conditional approval	./✗	./✗	./✗	./✗	✗/✗	./✗	./✗	✓/✗	./✗	./✗
Bosulif	Bosutinib	Conditional approval	✓/✓	./✓	./✗	✓/✓	✓/✓	✓/✓	./✗	✗/✓	✓/✓	./✗
Ceplene	Histamine dihydrochloride	Exceptional circumstances	./✗	./✗	./✗	./✗	./✗	./✗	./✗	./✗	./✗	./✗
Cometriq	Cabozantinib	Conditional approval	./✗	./✗	./✗	./✗	./✗	./✗	./✗	✗/✗	./✗	./✗
Dacogen	Decitabine	Unconditional	./✗	./✗	./✗	./✗	./✗	./✗	./✗	./✗	✓/✓	./✗
Darzalex	Daratumumab	Conditional approval	./✗	./✗	./✗	./✗	./✗	./✗	./✗	✗/✗	./✗	✗/✗
Farydak	Panobinostat lactate anhydrous	Unconditional	./✗	./✗	./✗	./✗	./✗	./✗	./✗	✗/✗	./✗	./✗
Gazyvaro	Obinutuzumab	Unconditional	./✗	✓/✓	✓/✓	./✗	✓/✓	✓/✓	✓/✓	✓/✓	./✗	✓/✓
Gliolan	5-aminolevulinic acid hydrochloride	Unconditional	./✗	./✗	./✗	./✗	./✗	./✗	./✗	./✗	./✗	./✗
Iclusig	Ponatinib	Unconditional	./✗	./✗	./✗	./✗	✗/✗	./✗	./✗	✓/✗	./✗	✓/✓
Imbruvica	Ibrutinib	Unconditional	✓/✓	./✗	./✗	✓/✓	✓/✓	./✗	✓/✓	✗/✓	./✗	✓/✓
Imnovid^a^	Pomalidomide	Unconditional	./✗	./✗	✓/✓	./✗	./✗	./✗	./✗	✗/✗	./✗	✗/✗
Kyprolis	Carfilzomib	Unconditional	./✗	./✗	./✗	./✗	./✗	./✗	./✗	./✗	./✗	./✗
Lartruvo	Olaratumab	Conditional approval	./✗	./✗	./✗	./✗	./✗	./✗	./✗	./✗	./✗	./✗
Lenvima	Lenvatinib mesylate	Unconditional	./✗	./✗	./✗	./✗	./✗	./✗	./✗	./✗	./✗	./✗
Lynparza	Olaparib	Unconditional	✓/✓	./✓	./✗	✓/✓	./✗	./✗	✓/✓	✗/✓	./✗	./✗
Mepact	Mifamurtide	Unconditional	./✗	./✗	✓/✓	✓/✓	✗/✗	./✗	./✗	✗/✗	./✗	✗/✗
Nexavar	Sorafenib	Unconditional	✓/✓	✓/✓	✓/✓	✓/✓	✓/✓	./✗	✓/✓	✗/✓	./✗	✓/✓
Ninlaro	Ixazomib citrate	Conditional approval	./✗	./✗	./✗	./✗	./✗	./✗	./✗	./✗	./✗	./✗
Onivyde	Irinotecan hydrochloride trihydrate	Unconditional	./✗	./✗	./✗	./✗	./✗	./✗	./✗	./✗	./✗	./✗
Revlimid	Lenalidomide	Unconditional	./✗	./✓	✓/✓	✓/✓	✓/✓	✗/✗	✓/✓	✓/✓	./✗	✓/✓
Sprycel	Dasatinib	Unconditional	✓/✓	✓/✓	✓/✓	✓/✓	✓/✓	✓/✓	✓/✓	✓/✓	✓/✓	✓/✓
Tasigna	Nilotinib	Unconditional	✓/✓	✓/✗	✓/✓	✓/✓	✓/✓	✓/✓	✓/✓	✓/✓	✓/✓	✓/✓
Tepadina	Thiotepa	Unconditional	./✗	./✗	./✗	./✗	./✗	./✗	./✗	./✓	./✗	./✗
Thalidomide Celgene^b^	Thalidomide	Unconditional	./✗	./✗	./✗	✓/✓	✓/✓	./✗	✓/✓	✗/✗	./✓	./✗
Torisel	Temsirolimus	Unconditional	✓/✓	✓/✗	✓/✓	./✗	✓/✓	✗/✗	./✗	✗/✓	./✗	✓/✓
Unituxin	Dinutuximab	Unconditional	./✗	./✗	./✗	./✗	./✗	./✗	./✗	./✗	./✗	./✗
Venclyxto	Venetoclax	Conditional approval	./✗	./✗	./✗	./✗	./✗	./✗	./✗	./✗	./✗	./✗
Vidaza	Azacitidine	Unconditional	✓/✓	✓/✗	✓/✓	✓/✓	./✗	./✗	✓/✓	✓/✓	./✗	✓/✓
Votubia	Everolimus	Unconditional	✓/✓	✓/✗	./✗	✓/✓	./✗	./✗	✓/✓	✓/✓	✓/✓	./✗
Xaluprine^c^	6-mercaptopurine monohydrate	Unconditional	✓/✓	./✗	✓/✓	✓/✓	✓/✓	./✗	./✗	./✗	./✗	./✗
Yondelis	Trabectedin	Unconditional	./✗	✓/✗	./✗	./✗	✗/✗	✗/✗	./✗	✓/✓	✓/✓	./✗
Zalmoxis	Allogeneic T cells genetically modified	Conditional approval	./✗	./✗	./✗	./✗	./✗	./✗	./✗	./✗	./✗	./✗

Scheme: recommendation/reimbursement

✓—positive; ✗—negative;.—not issued

^a^Previously pomalidomide celgene

^b^Previously thalidomide pharmion

^c^Mercaptopurine nova laboratories

## Recommendations and reimbursement status

Of all analysed countries, the highest share of positive recommendations was observed for Bulgaria and Estonia and the lowest, for Latvia. Negative recommendations were issued only in Hungary, Latvia, Poland, and Slovakia. The remaining countries issued only positive recommendations or no recommendation at all, which was due to specific regulations in those countries (Table 2).

**Table 2:** Share of positive recommendations and reimbursement decisions in analysed countries with respect to the type of approval

Country	Approval type	Recommendation			Reimbursement		Total
		Positive	Negative	No recommendation	Reimbursed	Not reimbursed	
Bulgaria	Conditional approval	2 (22.22%)	0 (0%)	7 (77.78%)	2 (22.22%)	7 (77.78%)	9
	Exceptional circumstances	1 (50%)	0 (0%)	1 (50%)	1 (50%)	1 (50%)	2
	Unconditional	10 (40%)	0 (0%)	15 (60%)	10 (40%)	15 (60%)	25
	Total	13 (36.11%)	0 (0%)	23 (63.89%)	13 (36.11%)	23 (63.89%)	36
Croatia	Conditional approval	1 (11.11%)	0 (0%)	8 (88.89%)	2 (22.22%)	7 (77.78%)	9
	Exceptional circumstances	0 (0%)	0 (0%)	2 (100%)	0 (0%)	2 (100%)	2
	Unconditional	9 (36%)	0 (0%)	16 (64%)	6 (24%)	19 (76%)	25
	Total	10 (27.78%)	0 (0%)	26 (72.22%)	8 (22.22%)	28 (77.78%)	36
Czechia	Conditional approval	0 (0%)	0 (0%)	9 (100%)	0 (0%)	9 (100%)	9
	Exceptional circumstances	1 (50%)	0 (0%)	1 (50%)	0 (0%)	2 (100%)	2
	Unconditional	10 (40%)	0 (0%)	15 (60%)	10 (40%)	15 (60%)	25
	Total	11 (30.56%)	0 (0%)	25 (69.44%)	10 (27.78%)	26 (72.22%)	36
Estonia	Conditional approval	2 (22.22%)	0 (0%)	7 (77.78%)	2 (22.22%)	7 (77.78%)	9
	Exceptional circumstances	0 (0%)	0 (0%)	2 (100%)	0 (0%)	2 (100%)	2
	Unconditional	11 (44%)	0 (0%)	14 (56%)	11 (44%)	14 (56%)	25
	Total	13 (36.11%)	0 (0%)	23 (63.89%)	13 (36.11%)	23 (63.89%)	36
Hungary	Conditional approval	2 (22.22%)	1 (11.11%)	6 (66.67%)	2 (22.22%)	7 (77.78%)	9
	Exceptional circumstances	1 (50%)	0 (0%)	1 (50%)	1 (50%)	1 (50%)	2
	Unconditional	9 (36%)	4 (16%)	12 (48%)	9 (36%)	16 (64%)	25
	Total	12 (33.33%)	5 (13.89%)	19 (52.78%)	12 (33.33%)	24 (66.67%)	36
Latvia	Conditional approval	1 (11.11%)	0 (0%)	8 (88.89%)	1 (11.11%)	8 (88.89%)	9
	Exceptional circumstances	0 (0%)	1 (50%)	1 (50%)	0 (0%)	2 (100%)	2
	Unconditional	3 (12%)	3 (12%)	19 (76%)	3 (12%)	22 (88%)	25
	Total	4 (11.11%)	4 (11.11%)	28 (77.78%)	4 (11.11%)	32 (88.89%)	36
Lithuania	Conditional approval	0 (0%)	0 (0%)	9 (100%)	0 (0%)	9 (100%)	9
	Exceptional circumstances	0 (0%)	0 (0%)	2 (100%)	0 (0%)	2 (100%)	2
	Unconditional	10 (40%)	0 (0%)	15 (60%)	10 (40%)	15 (60%)	25
	Total	10 (27.78%)	0 (0%)	26 (72.22%)	10 (27.78%)	26 (72.22%)	36
Poland	Conditional approval	2 (22.22%)	3 (33.33%)	4 (44.44%)	2 (22.22%)	7 (77.78%)	9
	Exceptional circumstances	1 (50%)	0 (0%)	1 (50%)	1 (50%)	1 (50%)	2
	Unconditional	8 (32%)	9 (36%)	8 (32%)	12 (48%)	13 (52%)	25
	Total	11 (30.56%)	12 (33.33%)	13 (36.11%)	15 (41.67%)	21 (58.33%)	36
Romania	Conditional approval	1 (11.11%)	0 (0%)	8 (88.89%)	1 (11.11%)	8 (88.89%)	9
	Exceptional circumstances	1 (50%)	0 (0%)	1 (50%)	1 (50%)	1 (50%)	2
	Unconditional	6 (24%)	0 (0%)	19 (76%)	7 (28%)	18 (72%)	25
	Total	8 (22.22%)	0 (0%)	28 (77.78%)	9 (25%)	27 (75%)	36
Slovakia	Conditional approval	0 (0%)	1 (11.11%)	8 (88.89%)	0 (0%)	9 (100%)	9
	Exceptional circumstances	0 (0%)	0 (0%)	2 (100%)	0 (0%)	2 (100%)	2
	Unconditional	10 (40%)	2 (8%)	13 (52%)	10 (40%)	15 (60%)	25
	Total	10 (27.78%)	3 (8.33%)	23 (63.89%)	10 (27.78%)	26 (72.22%)	36

Unconditional means that neither conditional approval nor exceptional circumstances were granted

The share of reimbursed drugs ranged from 11.11% in Latvia to 41.67% in Poland, with an average value of 28.89% (Table 2).

## Recommendations and reimbursement status in the context of the type of authorisation

The share of positive recommendations among conditionally approved drugs ranged from 0% in Czechia, Lithuania, and Slovakia to 22.22% in Bulgaria, Estonia, Hungary, and Poland. Considering exceptional circumstances, the share of positive recommendations ranged from 0% in Croatia, Estonia, Latvia, Lithuania, and Slovakia to 50% in Bulgaria, Czechia, Hungary, Poland, and Romania. Among drugs with no additional approval conditions, the lowest share of positive recommendations was observed in Latvia (12%), and the highest, in Estonia (44%) (Table 2).

## Agreement between recommendations and reimbursement status

In Hungary, of all 36 analysed drugs, 17 had both a recommendation and reimbursement decision issued. All drugs with a negative recommendation were not reimbursed and all drugs with a positive recommendation were reimbursed, resulting in perfect agreement with a kappa coefficient of 1 [95% CI 1–1]. A similar situation was observed in Latvia where of all 36 analysed drugs 8 had both a recommendation and reimbursement status available. All 4 drugs with a negative recommendation received no reimbursement, and 4 drugs with a positive recommendation received reimbursement, resulting in perfect agreement with a kappa coefficient of 1 [95% CI 1–1]. Slovakia demonstrated perfect agreement (kappa coefficient of 1; 95% CI 1–1) due to all 10 drugs with a positive recommendation being reimbursed and all 3 drugs with a negative recommendation not being reimbursed. However, it was different in Poland where 23 out of 36 drugs were analysed and the kappa coefficient was 0.4 [95% CI 0.04–0.76]. This is because out of 11 drugs with a positive recommendation 2 did not receive reimbursement, and out of 12 drugs with a negative recommendation, 5 were finally reimbursed.

For the purpose of the additional analysis (i.e. sensitivity analysis), the lack of HTA recommendation was treated as the third category (in addition to positive and negative recommendations), which allowed for the calculation of weighted kappa coefficients for all countries, with the same set of drugs analysed in each country. The sensitivity analysis revealed that the agreement was 0.42 [95% CI 0.33–0.53] for Bulgaria, 0.14 [95% CI 0.02–0.27] for Croatia, 0.35 [95% CI 0.23–0.47] for Czechia, 0.42 [95% CI 0.31–0.53] for Estonia, 0.46 [95% CI 0.33–0.58] for Hungary, 0.20 [95% CI 0.05–0.35] for Latvia, 0.36 [95% CI 0.24–0.48] for Lithuania, 0.30 [95% CI 0.13–0.46] for Poland, 0.29 [95% CI 0.16–0.42] for Romania and 0.39 [95% CI 0.26–0.51] for Slovakia.

## Public payer expenses for reimbursement of analysed orphan drugs

Total expenditures from the public budget on the reimbursement of oncology orphan drugs varied between countries and years. The expenditures ranged from almost 850 thousand euro in 2014 in Latvia to almost 75 million euro in 2016 in Poland. In all analysed countries, total expenses increased from 10% in Estonia to 243% in Lithuania between the years 2014 and 2016, with an average increase of 68%, as compared with an increase of only 8.5% in GDP and 9.3% in GDP per capita (Table 3).

**Table 3:** Total expenditures from public budget on the reimbursement of oncology orphan drugs

Country	2014 (million euro)	2015 (million euro)	2016 (million euro)
Bulgaria	11	13	16
Croatia	9	11	15
Czechia	27	36	44
Estonia	4	5	5
Hungary	18	27	41
Latvia	1	1	1
Lithuania	2	3	4
Poland	57	67	75
Romania	24	27	31
Slovakia	23	27	33

**Fig. 1:**
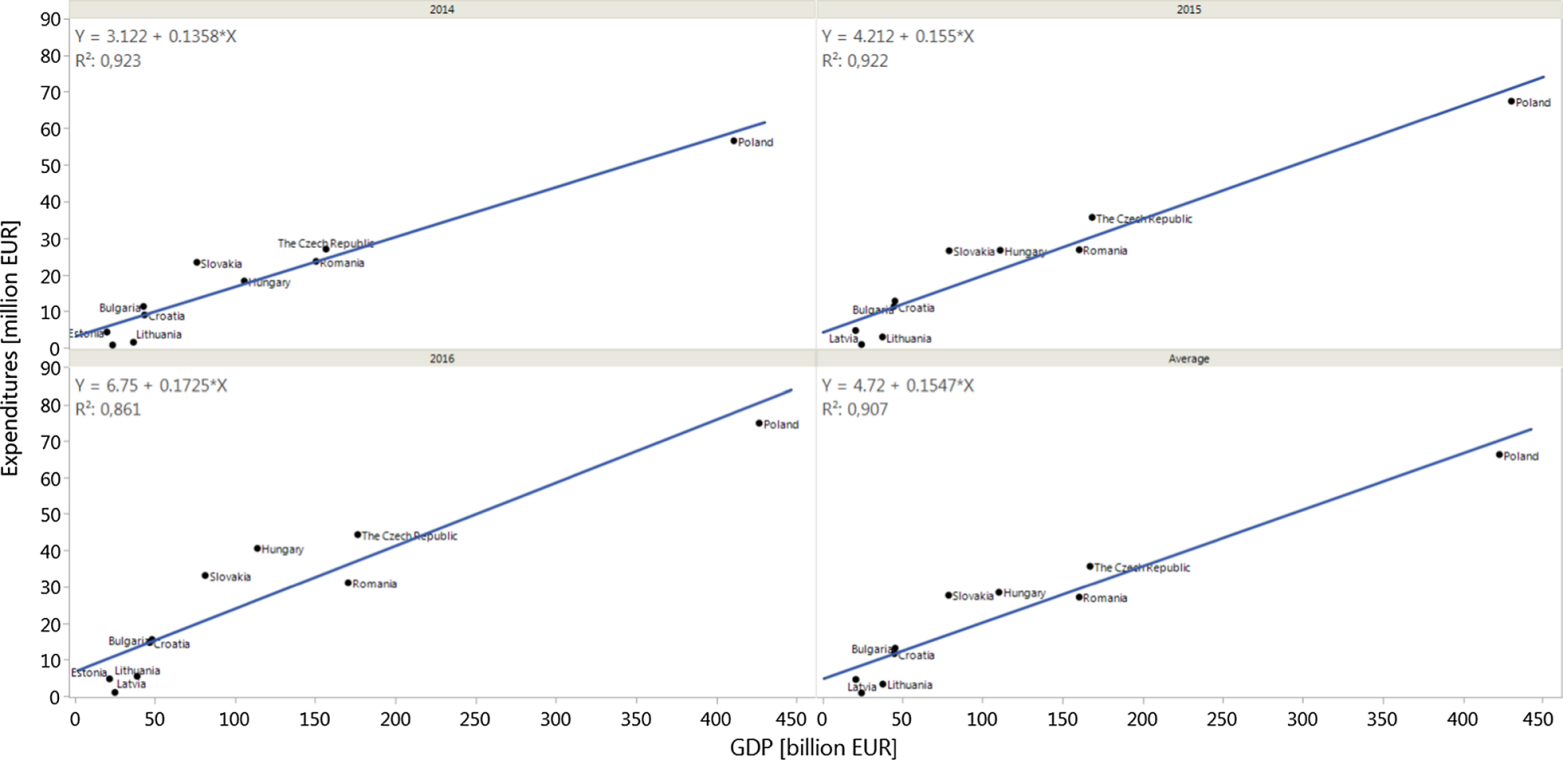
Correlation between total gross domestic product (GDP) and total public expenditures on the reimbursement of analysed drugs in 2014, 2015, and 2016, as well as an average value

Total expenditures on the reimbursement of analysed drugs were highly correlated with the total GDP in all countries, with a Pearson’s correlation coefficient of 0.961 [95% CI 0.838–0.99] in 2014, 0.96 [95% CI 0.836–0.99] in 2015, 0.93 [95% CI 0.72–0.98] in 2016, and an average coefficient of 0.95 [95% CI 0.81–0.99]. All correlations were significant, with p-values of less than 0.0001 (Fig. 1).

**Fig. 2:**
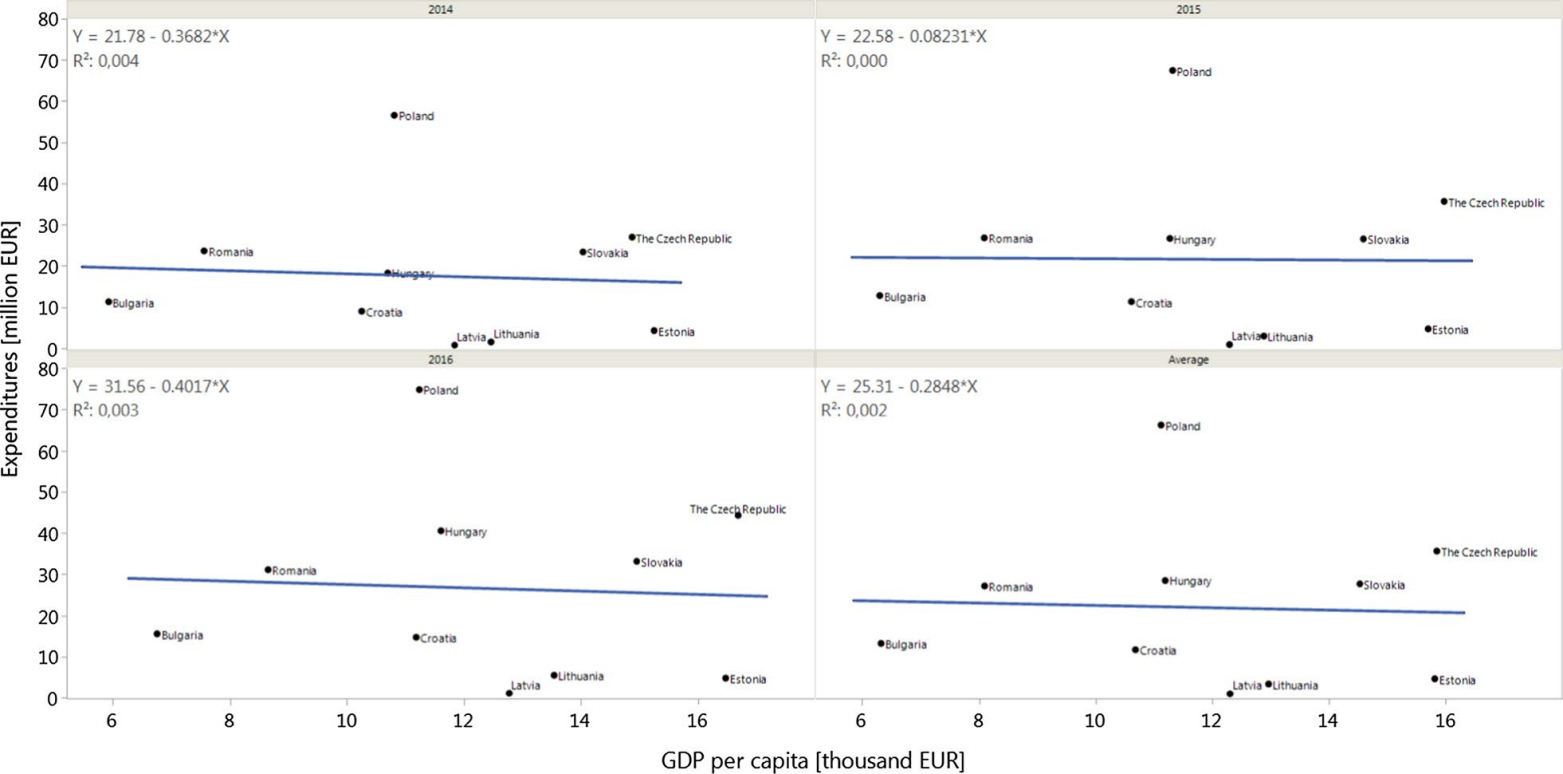
Correlation between total gross domestic product per capita (GDP per capita) and total public expenditures on the reimbursement of analysed drugs in 2014, 2015, and 2016, as well as an average value

**Fig. 3:**
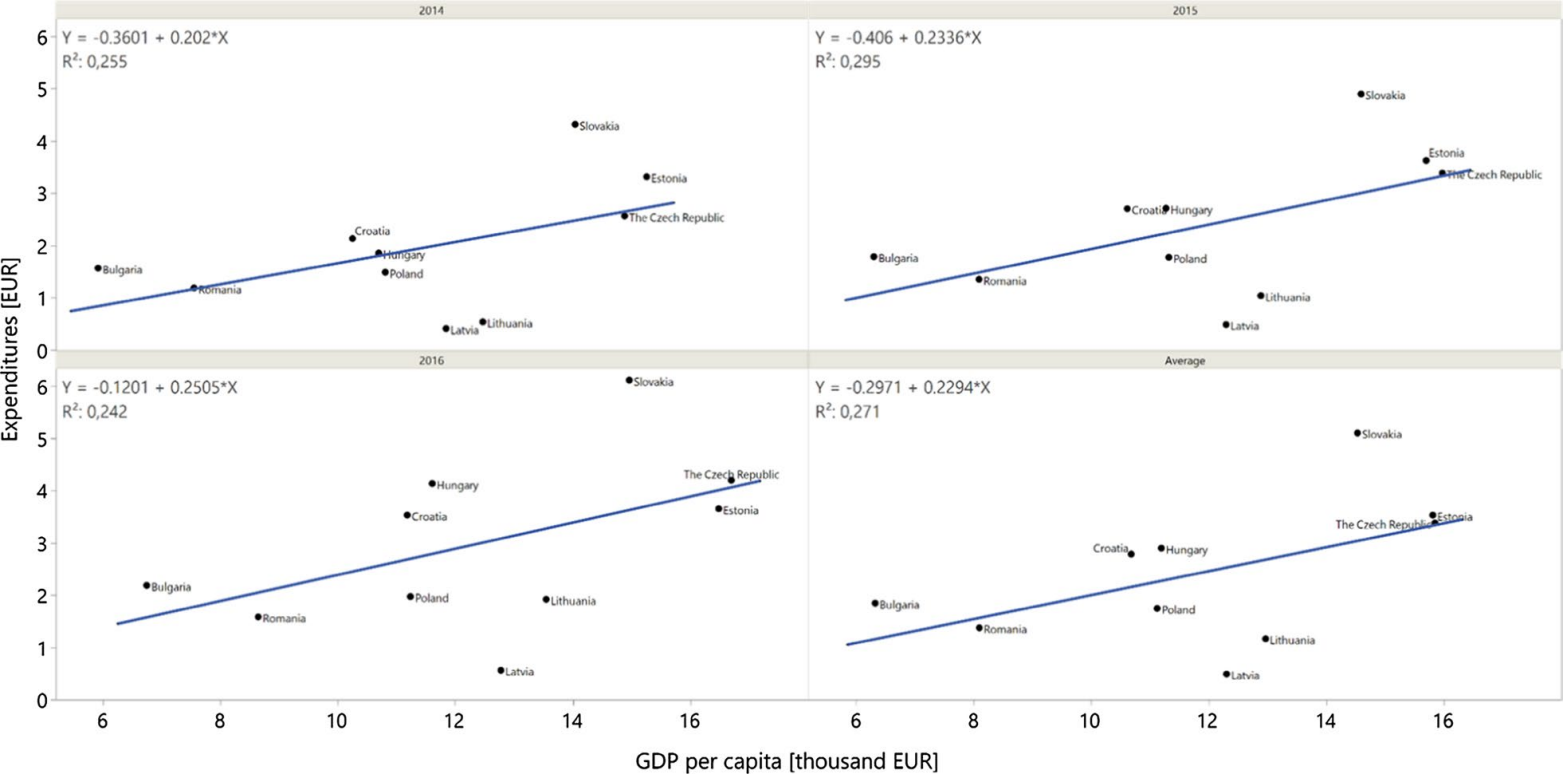
Correlation between total gross domestic product per capita (GDP per capita) and total public expenditures on the reimbursement of analysed drugs per capita in 2014, 2015, and 2016, as well as an average value

No significant correlations were observed for GDP per capita (Pearson’s correlation coefficient: − 0.07 [95% CI − 0.67 to 0.59] in 2014, − 0.01 [95% CI − 0.64 to 0.62] in 2015, − 0.06 [95% CI − 0.66 to 0.59] in 2016, and an average of − 0.04 [95% CI − 0.66 to 0.6]) (Fig. 2). Moderate, non-significant correlations were observed between GDP per capita and total expenditures per capita with values of 0.5048 [95% CI − 0.1830 to 0.8608; *p* = 0.14) in 2014, 0.5427 [95% CI − 0.1320 to 0.8738; *p* = 0.11) in 2015, 0.4923 [95% CI − 0.1991 to 0.8564; *p* = 0.15) in 2016, and an average coefficient of 0.5206 [95% CI − 0.1522 to 0.8663; *p* = 0.12) (Fig. 3). Moreover, no significant correlations were observed when analysing the share of reimbursed drugs (Pearson’s correlation coefficient: 0.536 [95% CI − 0.141 to 0.872] in 2014, 0.54 [95% CI − 0.136 to 0.873] in 2015, 0.536 [95% CI − 0.141 to 0.872] in 2016, and an average of 0.54 [95% CI − 0.136 to 0.873]). No correlation between GDP and the share of reimbursed orphan drugs was observed for analysed countries [12].

## Recommendations and HTA policy in analysed countries

In Bulgaria, during the time of the observation, recommendations on the reimbursement of a specific orphan drug are issued by the HTA Commission, the situation was then updated and now the National council of pricing and reimbursement is performing the procedure for HTA evaluation, pricing and inclusion in to the PDL. A positive recommendation from the commission is one of the conditions for reimbursement. The final decision on reimbursement and inclusion in the Positive Drug List is made by the National Council for Prices and Reimbursement of Medicinal Products. Another obligatory condition is to sign a managed entry agreement with the payer. Only the positive reports of the commission are published (on the website of the National Centre of Public Health and Analyses, along with full HTA reports). Orphan drugs to be reimbursed do not necessarily need to show cost-effectiveness, the additional assessments are also considered. HTA requirements include the results from clinical, pharmacoeconomic, and budget impact analyses, as well as ethical considerations. There is no need for an additional analysis. Bulgarian regulation on HTA necessitate for the applying products not to have negative HTA evaluation in UK, or France, or Germany. However nowadays it has changed and it is necessitate to have positive evaluation in UK, or France, or Germany, or Sweden (Table 4).

**Table 4:** Summary of aspects related to health technology assessment (HTA)

Question	Bulgaria	Croatia	Czechia	Estonia	Hungary	Latvia	Lithuania	Poland	Romania	Slovakia
Are there any advisory bodies (e.g. HTA agencies) that make recommendations whether or not to reimburse a specific orphan drug?	Yes	Yes	Yes	No	Yes	Yes	N/A	Yes	Yes	Yes
Does a positive recommendation mean the orphan drug will definitely be reimbursed?	No	Yes	Yes	No	No	No	Yes	No	Yes	No
Are the reimbursement recommendations publicly available?	Yes	No	Yes	Yes	No	Yes	No	Yes	Yes	Yes
Does the orphan drug need to show cost-effectiveness to be reimbursed?	No	Yes	Yes	Yes	Yes	Yes	Yes*	Yes	No	Yes
Does the orphan drug need to show an acceptable safety profile to be reimbursed?	Yes	Yes	Yes	Yes	Yes	Yes	Yes	Yes	No	Yes
Does the orphan drug need to show acceptable efficacy to be reimbursed?	Yes	Yes	Yes	Yes	Yes	Yes	Yes	Yes	No	Yes
Are there any HTA requirements for orphan drugs to be reimbursed?	Yes	Yes	Yes	Yes	Yes	No	N/A	Yes	N/A	Yes

*Not required for ultra-rare diseases; N/A – not applicable

In Croatia, most of the recommendations for orphan drugs are solved by the Croatian Health Insurance Fund (CHIF)’s Committee for Medicines, but HTA Agency can make a recommendation on the reimbursement of a specific orphan drug on special request. A positive recommendation of the CHIF’s Committee for Medicines means that the orphan drug will definitely obtain reimbursement. The CHIF’s list of the reimbursed drugs is publicly available, but it does not explain the procedure itself. The recommendations for reimbursement are internal documents of the CHIF. HTA requirements include results from sensitivity analysis, modelling, subgroup analysis, and others (Table 4).

In Czechia, the State Institute for Drug Control (SUKL) acts as an advisory and a decision-making body. The recommendations are published on the website, along with administrative files that can be accessed only with an electronic signature. All recommendations and files are available only in Czech. In most situations, orphan drugs must show cost-effectiveness in order to be reimbursed, apart from highly innovative medicinal products. In general, the same reimbursement rules apply to both orphan and non-orphan drugs and include acceptable efficacy, safety profile, and HTA requirements (Table 4).

In Estonia, there is no separate HTA advisory body; however, in some situations, the Ministry of Social Affairs and Estonian Health Insurance Fund ask the University of Tartu for advice, as it has a working group that writes HTA reports on special request. The reimbursement decision depends primarily on the budget impact, and a positive recommendation by the University of Tartu (if requested) does not guarantee reimbursement. The recommendations are published quarterly at the website of the Ministry of Social Affairs, or once a year at the website of the Health Insurance Fund for health care services. The HTA requirements for orphan drugs are the same as for other drugs (Table 4).

In Hungary, several advisory bodies make HTA recommendations, including the National Institute of Health Insurance Fund Management, Department of Health Technology Assessment, HTA Committee, Ministry of Human Capacities, and National Pharmaceutical Therapeutic Committee. A positive recommendation by any of those bodies does not always translate into a positive reimbursement decision, because the reimbursement process is more complex. For new active substances or a new indication, it is necessary to amend the reimbursement law. Only the final decisions, but not recommendations, are published. The most important criteria for reimbursement are cost-effectiveness, equity, budget impact, the severity of illness, and efficacy. The acceptable safety profile is analysed before the marketing authorisation by the EMA or the National Institute of Pharmacy and Nutrition. The evaluation by an HTA department includes the analysis of the safety profile (e.g. considering real-world evidence, among other important factors). There are no separate HTA requirements for orphan drugs (Table 4).

In Latvia, decisions on reimbursement are made by the National Health Service (NHS). Internal National Health Service bodies make recommendations, which are published on the website. Inclusion in a positive drug list is possible if the drug is cost-effective, has no budget impact, or if additional financial resources are allocated for this aim (Table 4).

In Lithuania, there is no stand alone HTA body. However, therapeutic value of medicines applied for reimbursement is assessed by State Medicines Control Agency, whereas economic evaluation is carried out by the Pharmaceutical department of the Ministry of Health. Decision on reimbursement takes Reimbursement Commission of the Ministry of Health taking into account therapeutic and economical evaluation. Assessment reports of therapeutic value and economical evaluation as well as protocols of the meetings of the reimbursement commission are publicly available on the website of the Ministry of Health (in Lithuanian language). Drugs are reimbursed if they are included in the reimbursement list. Each drug to be included in a positive drug list must show cost-effectiveness, except for orphan drugs for ultra-rare diseases. Conditional approval or approval under exceptional circumstances are not specifically taken into account. Decision is made on evidence of clinical value (Table 4).

In Poland, there are 2 advisory bodies: the Agency for Health Technology Assessment and Tariff System (AOTMiT) and within AOTMiT the Transparency Council. The AOTMiT makes positive or negative recommendations. The Transparency Council issues opinions to the President of the AOTMiT that can also be positive or negative. Neither positive recommendation by AOTMiT nor TC guarantees reimbursement. All recommendations and opinions are publicly available at the AOTMiT’s website (Table 4).

In Romania, the National Agency for Medicines and Medical Devices is an advisory body in the reimbursement decision-making process. A drug is reimbursed following the positive advice of the agency; however, new drugs that require additional funding are subject to volume price negotiations. Orphan drugs that are included in a national therapeutic programme are fully reimbursed. Once a drug receives a positive recommendation, it must be officially included in the list and the publicly available guideline for a specified disease is modified. Then, the drug is reimbursed by the Ministry of Health and National Agency for Medicines and Medical Devices (NHIH) (Table 4).

The Slovak Ministry of Health established the Reimbursement (or Categorisation) Committee to act as its advisory body with regards to reimbursement. The committee is supported by different advisory working groups, a medical board (assessing the effectiveness, safety, and importance of the medicine), and the Working Group for Pharmacoeconomics, Clinical Outcomes and Health Technology Assessment of the Ministry of Health. The recommendation of the Categorisation Committee can be overruled by the Minister of Health. Recommendations are publicly available at the website of the ministry. Orphan drugs must show cost-effectiveness; however, the thresholds were not applicable for orphan drugs indicated in the therapy of rare diseases with a prevalence of less than 1:100,000 in Slovakia. Based on the evaluation of the drug’s effectiveness, safety, and importance as well as economic benefits, the Categorisation Committee determines its therapeutic and social value. Again, the thresholds were not applicable for orphan drugs indicated in the therapy of rare diseases with a prevalence of less than 1:100,000. The threshold prevalence was updated in 2018 to 1:50,000 (Table 4).

## Discussion

The study revealed that most analysed countries implemented some sort of an HTA process in their reimbursement decision-making process and in the majority of these countries, publicly available reimbursement recommendations were used.

In general, in all analysed countries orphan drugs are required to be cost-effective, present an acceptable safety profile and high enough efficacy however different rules are applied when making final decision. Although the recommendations do not easily translate into positive reimbursement decision the observed kappa coefficients were high. The study showed that, in most countries, HTA recommendations are issued together with positive reimbursement decisions, which translates into a perfect agreement of 1. The exception was Poland, where the kappa coefficient was 0.4, mainly because almost 42% of drugs with a negative recommendation were finally reimbursed. Importantly, once the reimbursement recommendation is issued by the AOTMiT in Poland, the Marketing Authorisation Holder can enter negotiations with the Ministry of Health. This often results in reducing the cost of the drugs or introducing risk-sharing schemes, which has a direct impact on reimbursement. Unlike the recommendations by the AOTMiT, the negotiations are not publicly available and thus cannot be analysed.

In addition, we assessed the burden of costs generated by the reimbursement of the analysed oncology drugs in the years 2014–2016 on the public payer budget. An average increase in public expenditures on the reimbursement was 68%, as compared with an increase of only 8.5% in the total GDP and of 9.3% in GDP per capita. The factors influencing the increase could be associated with an increased number of reimbursed drugs, subject that was covered extensively by Vokinger and Kesselheim [11] as well as changes in public budget expenditures or in reimbursement policy. Changes in pricing could also result in fluctuations in expenditures. In most cases, after the initial approval of the drug, some new clinical data regarding drug efficacy would emerge and could influence reimbursement decisions. Although the Committee of Human Medicinal Products does not have any direct influence on prising policy in European Union (EU) member states, the type of approval could be considered in the decision-making process [5]. Additionally, in this study, we focused on the analysis of growing expenditures in relation to growing GDP without considering the effect of various factors on the expenditures to reveal the burden of oncology orphan drugs on the public budget. Public expenditures were significantly correlated with the total GDP but not with GDP per capita or the share of reimbursed oncology orphan drugs, which might indicate that the reimbursement of oncology orphan drugs could be associated with the general size of country economics rather than the welfare of its citizens. This could result from a policy that is implemented by many countries, namely, to reduce expenses on public health to the country’s GDP and not GDP per capita. The most informative correlation between GDP per capita and expenditures per capita was moderate in size, however statically insignificant.

To our knowledge, this is the first study to summarise HTA decision making regarding oncology orphan drugs in EU countries from the CEE region, as well as to analyse the dynamics of public expenditures on the reimbursement of those drugs in relation to the GDP and GDP per capita.

We were not able to collect relevant data for Slovenia, which is a limitation of the study. In addition, we used the kappa coefficient to analyse the agreement between recommendations and reimbursement status. However, the kappa coefficient of agreement could be calculated only for the countries that issued both positive and negative recommendations to provide some insight into the functioning of the reimbursement policy based on HTA-assessed drugs. Moreover, the coefficient might have been affected by the levels of analysed variables, and thus it can be treated as a descriptive rather than inferential statistics. As in other statistical tests, only cases (drugs) with both the recommendation and reimbursement status available were analysed, which resulted in different sets of drugs used to calculate kappa coefficients in different countries, this approach is however appropriate as using in analysis drugs that were not even considered for heath technology assessment could introduce bias into the analysis. Also, we observed that most kappa coefficients in this study were equal to 1. This could result from the fact that the countries significantly differed in terms of HTA processes, which translates into a considerable discrepancy in the shares of positive and negative recommendations. In some countries, advisory bodies issued only positive recommendations.

Another limitation is that the study focused on a set of oncology orphan drugs only in one year. Further research is needed to compare oncology orphan drugs to non-oncology ones (e.g. metabolic) and to analyse costs over a longer period.

In order to compare the results of our study, we reviewed medical databases to identify other important publications on this subject. Our previous study [5] analysed the status of all drugs with orphan designation and their relation to the type of EMA approval. In addition, we showed a significant variation in agreement with the reimbursement status of analysed drugs across selected European countries. The same list of orphan drugs was used in another study [12] that described the status of orphan drugs in CEE-EU countries. The reimbursement of orphan drugs was assessed without describing detailed HTA or public budget issues. Both studies showed a significant impact of the type of approval and disease on the reimbursement decisions. Moreover, they revealed that the shares of reimbursed orphan drugs are much higher in Western European than CEE-EU countries. In addition, we showed that the type of the disease was significantly associated with the type of marketing authorisation. We also reported that oncology drugs were significantly associated with the chances of reimbursement. For example, in Croatia, oncological orphan drugs were more than 5 times more likely to be reimbursed compared with non-oncology drugs (OR 5.33; 95% CI 1.31–21.68).

The field of oncology orphan drugs was examined in a research by Jarosławski et al. and revealed cost differences between oncology orphan drugs targeted at smaller populations and those targeted at larger populations in the United States [13]. No similar research was conducted for Europe. On the other hand Vassel et al. analysed whether children and adolescents with cancer benefited from the Orphan Drug Regulation in the EU and showed that only 2% of oncology orphan drugs were designed for use by children. The analysis covered the period from 8 August 2000 to 10 September 2016 [15].

The subject of oncology orphan drugs is not yet well described, especially among CEE countries, which struggle with a growing burden of reimbursement for the public budget and which need detailed data for efficient reimbursement decision making and HTA assessment regarding orphan drugs (particularly those for oncological diseases).

## Conclusions

In Hungary, Latvia, and Slovakia, a positive recommendation was associated with a positive reimbursement decision, while a negative recommendation, with a lack of reimbursement. The reimbursement of oncology orphan drugs is associated with a growing burden for the public budget, with an average 3-year increase in expenses of 68%, as compared with an increase of only 8.5% in the total GDP and of 9.3% in GDP per capita among CEE countries. The total expenditures on the reimbursement of oncology orphan drugs varied among countries and were highly correlated with the total national GDP but not with GDP per capita. Expenditures per capita also were not significantly correlated with GDP per capita. The highest share of drugs with any recommendation was observed in Poland, and the lowest, in Latvia and Romania. The share of reimbursed drugs was the lowest in Latvia and the highest in Poland.

## Methods

In a previous study [5] we reviewed the EMA website [1] and identified all drugs with orphan designation in 2017. We also collected data on the type of approval, disease, and reimbursement status [4] for Bulgaria, Croatia, Czechia, Estonia, Hungary, Latvia, Lithuania, Poland, Romania, and Slovakia. For the current study, we selected only drugs used in the treatment of oncological diseases (38% of all analysed drugs) and performed a questionnaire survey among experts from respective countries. We collected additional data on total public expenditures on the reimbursement of those drugs as well as HTA recommendations issued for those drugs by reimbursement advisory bodies operating in the analysed countries. As the economic burden generated for national public payers reflects real cash flows in the years 2014 to 2016, no inflation corrections were made. Local currency units were converted to euros. The total expenditures per year per drug per country included the cost of reimbursement, expenditures for individual request, and expenditures of partial reimbursement of specific drugs with patients’ co-payment. The costs were presented from the perspective of health care system; no distinction was made on whether the cost of drug was totally covered by the public payer or was a patients’ co-payment involved. Only budget impact was analysed which took into account real cash flows from the public payer hence the data has been already corrected for any existing risk sharing agreements or drug price differences [12]. The correlation between the total cost (as well as per capita) and gross domestic product (GDP) and GDP per capita was analysed using Pearson’s correlation coefficient. Data on GDP in US dollars for each analysed year were derived from the World Bank [9] and were converted in euro using the following exchange rates: 0.7536 in 2014, 0.9017 in 2015, and 0.9042 in 2016.

Nominal variables were presented as counts and percentages. Cost data were rounded to units in euros. The agreement between a reimbursement recommendation and status in the CEE countries was assessed using the κ coefficient [10]. All κ coefficients were supported with 95% confidence intervals (CIs) and rounded to 2 decimal places.

Statistical analyses were performed in the JMP® software, version 14.2.0 (SAS Institute Inc., 2018, Cary, North Carolina, USA).

## Data Availability

The datasets used and/or analysed during the current study are available from the corresponding author on reasonable request.

## Abbreviations

HTA: Health technology assessment
EMA: European Medicines Agency
GDP: Gross domestic product
CEE: Central and Eastern Europe
CI: Confidence interval
CHIF: Croatian Health Insurance Fund
NHIH: National Agency for Medicines and Medical Devices
AOTMiT: Agencja Oceny Technologii Medycznych i Taryfikacji
SUKL: State Institute for Drug Control
NHS: National Health Service
TC: Transparency Council
